# Association Between Diabetes Risk Reduction Diet and Lung Cancer Risk in 98,159 Participants: Results From a Prospective Study

**DOI:** 10.3389/fonc.2022.855101

**Published:** 2022-04-28

**Authors:** Yin Zhang, Guochao Zhong, Min Zhu, Ling Chen, Huajing Wan, Fengming Luo

**Affiliations:** ^1^Department of Respiratory and Critical Care Medicine, West China Hospital, Sichuan University, Chengdu, China; ^2^Laboratory of Pulmonary Immunology and Inflammation, Frontiers Science Center for Disease-related Molecular Network, West China Hospital, Sichuan University, Chengdu, China; ^3^Clinical Research Center for Respiratory Disease, West China Hospital, Sichuan University, Chengdu, China; ^4^Department of Hepatobiliary Surgery, The Second Affiliated Hospital of Chongqing Medical University, Chongqing, China

**Keywords:** diabetes risk reduction diet score, lung cancer, prevention, prostate lung colorectal and ovarian cancer screening trial, dose - response

## Abstract

**Purpose:**

To evaluate the association between diabetes risk reduction diet (DRRD) score and the risk of lung cancer in a large population.

**Methods:**

Data of participants in this study were collected from the Prostate, Lung, Colorectal, and Ovarian (PLCO) Cancer Screening Trial. Hazard ratios (HRs) and 95% confidence intervals (CIs) were calculated in the Cox proportional hazards regression model for the association of DRRD score and lung cancer incidence in all included participants. Prespecified subgroup analyses were performed to evaluate whether the observed association was modified by age, sex, BMI, race/ethnicity, family history of lung cancer, smoking status and history of diabetes.

**Results:**

A total of 98,159 participants were included in this study. The mean (SD) age of the study participants cohort at baseline was 65.5 (5.73) years old. The mean (SD) follow-up time was 8.83 (1.96) years. The mean (SD) score of DRRD was 26.82 (5.19), and ranged from 20.47 (2.3) to 33.65 (2.42) from the lowest quartile to the highest quartile of the DRRD score, inferring the possibility of highest through the lowest risk of type 2 diabetes. The calculated HRs showed there was a trend that higher quartile indicated lower risk of lung cancer after adjusted for covariates (HR_Q4vsQ1_: 0.85; 95% CI:0.73,0.98; p for trend =0.036). The inverse trend between higher DRRD score and the risk of squamous cell carcinoma was more evident (HR_Q4vsQ1_: 0.50; 95% CI:0.34,0.73; p for trend =0.002). The inverse association between DRRD score and the incidence of lung cancer was more pronounced in participants who had a clear family history of lung cancer (p for interaction=0.016).

**Conclusion:**

A protective association between DRRD score and risk of lung cancer is obtained. People are encouraged to adhere to higher DRRD score in their daily diet. Further studies should be conducted to confirm the result and explore the mechanism.

## Highlights

1. This prospective study explores the association between DRRD score and the incidence of lung cancer for the first time in a large population, with a long follow-up. The results encourage people to adhere to DRRD score in their daily diet.

2. We interestingly found that the inverse association between DRRD score and the incidence of lung cancer was more pronounced in participants with a clear family history of lung cancer, although the reason is still exactly unclear.

## Introduction

Lung cancer is the second most diagnosed malignancy worldwide and new cases of lung cancer accounted for 11.4% of all cancers in 2020 (1). It is presented as the leading cause of all cancer deaths, with an estimated 1.8 million deaths each year (1, 2). The association between diet and lung cancer has been studied by researchers globally and some conclusions have been subsequently reached. Evidence suggested specific dietary habits might influence lung cancer risk and play a role in lung cancer prevention. Dietary inflammatory index (DII), calculated to assess the inflammatory levels in one’s diet, has been implicated in the pathogenesis of lung cancer (3). In a population-based, prospective cohort followed up for 17.5 years, the risk of lung cancer was decreased by 10% in the highest quality group compared with the lowest one based on quintiles for DII (4). In an updated pooled analysis including five cohort studies (n = 12,730 incident cases), higher adherence to the Mediterranean diet, a plant-based pattern, has been reported to decrease the risk of lung cancer by 16% (5). Both patterns encouraged good diet quality to reduce inflammation levels in the human body (6, 7). In recent years, a dietary pattern for diabetes prevention named diabetes risk reduction diet (DRRD) was established (8). Nine dietary variables including cereal fiber, nuts, coffee, whole fruits, the ratio of polyunsaturated to saturated fat, glycemic index (GI), trans-fat, sugar-sweetened beverages (SSBs)/fruit juices, and red and processed meats composed of DRRD (8). Higher DRRD score was suggested to have the potential to reduce the possibility of hyperinsulinemia and insulin resistance (9), which were demonstrated to be closely related to increased cancer risk, including lung cancer (10–12). The mechanism may be associated with the activation of insulin-like growth factor-1, stimulating of the Ras signaling pathway, local angiogenesis or growth promotion *via* insulin receptors present on lung cancer cells (11, 13, 14). Therefore, we hypothesize that higher DRRD score may be inversely associated with lung cancer risk (15). This study aims to evaluate the association between DRRD score and the risk of lung cancer in a large population.

## Materials and Methods

### Study Population

Data of participants in this study were from the Prostate, Lung, Colorectal, and Ovarian (PLCO) Cancer Screening Trial, a large-scale randomized clinical trial (RCT) designed and sponsored by United States National Cancer Institute (NCI) to evaluate whether screening methods can reduce mortality from prostate, lung, colorectal, and ovarian cancers in men and women aged 55 to 74. The PLCO trial was carried out at 10 centers in the United States from 1993 to 2001, actually enrolling 154,000 healthy subjects between ages 55 and 74 at enrollment according to the eligibility requirements. The participants who met the eligibility criteria were randomized to the intervention group (received certain screening tests) or the control group (received usual care). All participants were asked to complete self-reported questionnaires about the lifestyles and followed up until 2009 for cancer incidence. The questionnaires included baseline questionnaire (BQ), diet history questionnaire (DHQ) (16). The BQ was given to participants to collect the baseline information at enrollment. Furthermore, the information about cancer diagnosis was also recorded in the BQ. The DHQ was a food frequency questionnaire (FFQ) containing dietary information, which evaluated the food or nutrients intake over the past year and took subjects about 1 h to complete. Several studies have assessed its validity, showing DHQ a good instrument for nutrients’ evaluation (17, 18).

According to the objective of this study, we excluded subjects if they: 1) failed to provide complete baseline information; 2) failed to complete a valid DHQ (i.e., a date of completion was not available; the date of completion was not prior to the date of death; there were at least 8 missing frequency responses; calorie intake was extreme (top 1% and bottom 1%) for each gender); 3) had a history of any cancer before DHQ entry. This study has been approved by the United States NCI (CDAS project “PLCO-800”). The written informed consent to participate in the study was provided by each participant, and the study protocol was approved by the Institutional Review Board of the United States NCI (https://biometry.nci.nih.gov/cdas/plco/).

### Data Collection

Participants were arranged to complete a self-administered BQ containing personal baseline information. In this study, we collected trial arm, age, body mass index (BMI), sex, marital status, race/ethnicity, smoking status, pack-years of cigarettes, family history of lung cancer, family history of any cancer, and history of diabetes. DHQ was used to collect dietary information, including alcohol intake, total energy intake, and intake of food or nutrients in diet to calculate DRRD score (see *DRRD Score Calculation*).

### DRRD Score Calculation

The intake of food and nutrients was collected in DHQ for each included participant. The participants completed DHQ at an average time of about 3 years after the randomization (https://epi.grants.cancer.gov/dhq/about/). DRRD score was the sum of the quintile values from 1 to 5 of 9 dietary variables including cereal fiber, nuts, coffee, whole fruits, and ratio of polyunsaturated to saturated fat (higher quintiles of intake/value indicate higher scores); and GI, trans-fat, SSBs/fruit juices, and red and processed meats (higher quintiles of intake/value indicate lower scores) (8). After the sum-up of the quintile values of the nine factors, the DRRD score ranged from 9 to 45, inferring the possibility of highest through the lowest risk of T2D. In this study, DRRD score was divided into quartiles. Baseline characteristics of participants were presented by quartile of DRRD score (quartile 1 to quartile 4).

### Lung Cancer Ascertainment

In this study, the end point was the incidence of lung cancer. In the PLCO trial, the identification of lung cancer was based on reports abstracted from the annual study update forms and then the diagnosis was confirmed in relevant medical records obtained through ICD-O codes and extracted using standardized forms. Of note, carcinoid lung cancer was not considered as a target of lung cancer screening in the PLCO trial, thus, it was not confirmed as lung cancer in this study.

### Statistical Analysis

Continuous variables were presented as mean (standard deviation), and categorical variables were presented as numbers (percentage). The Kruskal-Wallis test and chi-square test were used to compare continuous and categorical variables, across the groups of participants, respectively. Hazard ratios (HRs) and 95% confidence intervals (CIs) were calculated in the Cox proportional hazards regression model for the association of DRRD score and lung cancer incidence in all included participants. To test whether a trend existed across quartiles of DRRD score for the lung cancer risk estimation, the median value of each quartile was first assigned to each subject in the quartile and then treated as a continuous variable in the regression models, with the lowest quartile as the reference group. Sub-analyses were further performed to evaluate associations with different histological types including adenocarcinoma, squamous cell carcinoma, large cell carcinoma, and small cell carcinoma. Covariates included in the multivariate regression models were based on the literature review and clinical judgement. In detail, age (continuous), sex (male or female), BMI (continuous), total energy intake (continuous), family history of lung cancer (yes, no, or possible), marital status (married or not married), race/ethnicity (white or non-white), smoking status (never, former smoker, current smoker), pack-years of cigarettes (continuous), alcohol intake (never, former, current, or unknown), history of diabetes (yes or no) were adjusted as covariates. The dose-response analysis was conducted to explore the relationship between DRRD score and the incidence of lung cancer. A restricted cubic spline model with three knots at the 10th, 50th, and 90th percentiles was employed (19). We chose the mean value of DRRD score as the reference level (19). Prespecified subgroup analyses were performed to evaluate whether the observed association of DRRD score with lung cancer incidence was modified by age (>65 vs. ≤65 years old), sex (male vs. female), BMI (>25 vs. 25-30 vs. ≥30 kg/m^2^), race/ethnicity (white vs. non-white), family history of lung cancer (yes vs. no/possible), smoking status (non-smokers vs. smokers), and history of diabetes (yes vs. no). Furthermore, we conducted the sensitivity analysis to test the robustness of the results by excluding participants 1) with extreme energy intake (>4000 kcal/day or <500 kcal/day), 2) with extreme BMI (top 1% and bottom 1%), 3) with diabetes, and 4) with a follow-up less than 2 years. A two-tailed p value less than 0.05 was considered significant. The statistical analyses were conducted using STATA 15.1, SPSS 25.0, and R 3.6.1 software.

## Results

### Baseline Characteristics

**Figure 1:**
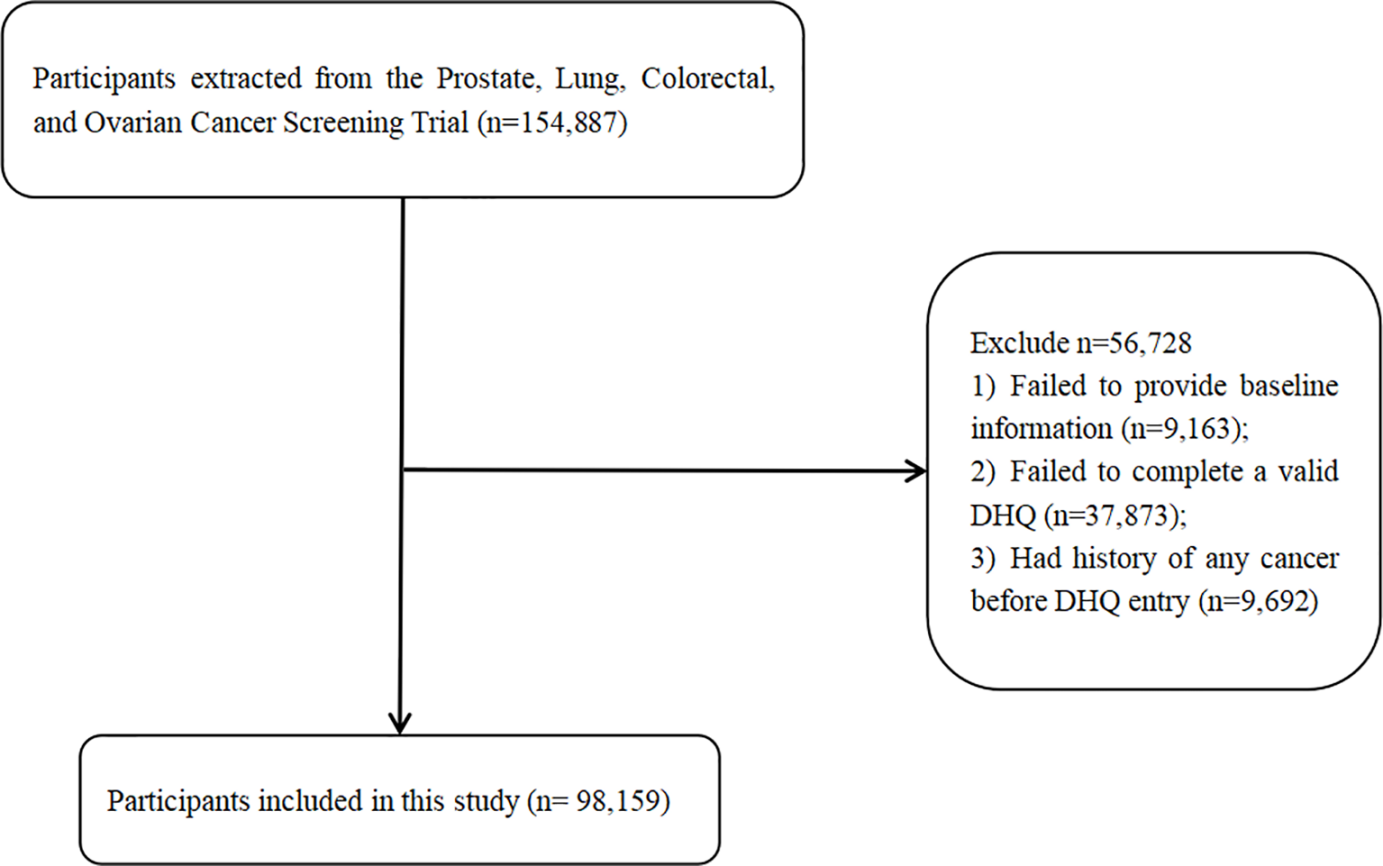
Flow diagram of the selected individuals.

Data of 98,159 participants were extracted after excluding the participants according to exclusion criteria. The detailed flow chart is presented in **Figure 1**. According to the DRRD score, we divided participants into quartiles (26,423 in Q1; 28,334 in Q2; 19,643 in Q3; and 23,759 in Q4). In the included population, the mean (SD) age of the study participants cohort at baseline was 65.5 (5.73) years old. The mean (SD) follow-up time was 8.83 (1.96) years. There were finally 50,316 (51.26%) participants enrolled in the intervention group and 47,843 (48.74%) participants recruited to the control group. Compared with participants in the lowest quartile group, those in the highest group had lower daily energy intake. They also had higher intake of fruit, nut or peanut butter, coffee, cereal fiber, and polyunsaturated fat to saturated fat, but lower intake of diets with higher glycemic index, trans fat, sugar-sweetened beverage/fruit juice, and red and processed meat. In summary, the mean (SD) score of DRRD was 26.82 (5.19) in all participants and ranged from 20.47 (2.3) to 33.65 (2.42) from the lowest quartile to the highest quartile of DRRD score. More younger, more male, more white, more married, and subjects with higher BMI were in the lowest DRRD quartile than in the highest quartile. There were more non-smokers and non-drinkers in the highest DRRD quartile than in the lowest quartile, while more pack-years of cigarettes was observed in the lower quartile of DRRD. More participants had a clear family history of lung cancer but fewer had a family history of any cancer in the highest DRRD quartile. More detailed information is shown in **Table 1**.

**Table 1:** Baseline characteristics of 98,159 participants from Prostate, Lung, Colorectal, and Ovarian Cancer Screening Trial by DRRD Quartiles.

	Overall	Quartile 1	Quartile 2	Quartile 3	Quartile 4	p-value*
**Number of participants**	98,159	26,423	28,334	19,643	23,759	–
**Number of cases**	1,632	555	482	281	314	–
**Person-years**	866776.8	230353.4	249861.1	174590.2	211972.0	–
**Follow-up, years**	8.83 (1.96)	8.72 (2.02)	8.82 (1.97)	8.89 (1.92)	8.92 (1.89)	<0.001
**Total energy intake, kcal/day**	1737.78 (734.7)	1806.45 (742.14)	1744.9 (787.74)	1703.73 (752.7)	1681.09 (632.23)	<0.001
**DRRD score**	26.82 (5.19)	20.47 (2.38)	25.54 (1.11)	28.94 (0.81)	33.65 (2.42)	<0.001
**Fruit, servings/day**	2.73 (2.04)	1.64 (1.18)	2.4 (1.68)	3.03 (1.96)	4.11 (2.38)	<0.001
**Nut or peanut butter, times/day**	6.72 (14.51)	4.24 (8.22)	5.82 (12.19)	7.19 (15.23)	10.18 (20.13)	<0.001
**Coffee, g/day**	842.66 (793.13)	725.83 (782.98)	860.39 (802.41)	887.58 (791.26)	914.32 (780.63)	<0.001
**Cereal fiber, g/day**	17.86 (8.42)	14.27 (6.2)	16.72 (7.68)	18.69 (8.36)	22.55 (9.16)	<0.001
**Ratio of polyunsaturated fat to saturated fat**	0.76 (0.26)	0.62 (0.18)	0.72 (0.22)	0.8 (0.24)	0.94 (0.28)	<0.001
**Glycemic index of diet**	53.55 (3.31)	55.57 (3.04)	53.9 (3.06)	52.81 (2.95)	51.5 (2.7)	<0.001
**Trans fat, g/day**	3.98 (2.39)	4.95 (2.52)	4.22 (2.49)	3.65 (2.22)	2.91 (1.65)	<0.001
**Sugar-sweetened beverage/fruit juice, g/day**	217.98 (391.18)	399.38 (529.15)	215.86 (359.09)	143.21 (281.31)	80.61 (203.77)	<0.001
**Red and processed meat, g/day**	12.4 (15.27)	19.73 (19.26)	13.48 (15.04)	9.57 (11.41)	5.31 (7.45)	<0.001
**Trial arm**						0.047
;Intervention	50316 (51.26%)	13472 (50.99%)	14564 (51.4%)	9948 (50.64%)	12332 (51.9%)	
Control	47843 (48.74%)	12951 (49.01%)	13770 (48.6%)	9695 (49.36%)	11427 (48.1%)	
**Age, years**	65.5 (5.73)	64.52 (5.57)	65.47 (5.68)	65.91 (5.76)	66.29 (5.77)	<0.001
**Sex**						<0.001
Female	50637 (51.59%)	10857 (41.09%)	13932 (49.17%)	10877 (55.37%)	14971 (63.01%)	
Male	47522 (48.41%)	15566 (58.91%)	14402 (50.83%)	8766 (44.63%)	8788 (36.99%)	
**Baseline body mass index, kg/m2**	27.23 (4.81)	28.22 (4.99)	27.52 (4.82)	26.93 (4.64)	26.01 (4.45)	<0.001
**Race/ethnicity**						<0.001
White	89341 (91.02%)	24285 (91.91%)	25897 (91.4%)	17889 (91.07%)	21270 (89.52%)	
Non-white	8818 (8.98%)	2138 (8.09%)	2437 (8.6%)	1754 (8.93%)	2489 (10.48%)	
**Marital status**						<0.001
Married	76967 (78.41%)	21151 (80.05%)	22595 (79.75%)	15439 (78.6%)	17782 (74.84%)	
Not married	21192 (21.59%)	5272 (19.95%)	5739 (20.25%)	4204 (21.4%)	5977 (25.16%)	
**Smoking status**						<0.001
Non-smokers	47395 (48.28%)	11835 (44.79%)	13353 (47.13%)	9744 (49.61%)	12463 (52.46%)	
Former smokers	41683 (42.46%)	11093 (41.98%)	12141 (42.85%)	8451 (43.02%)	9998 (42.08%)	
Current smokers	9081 (9.25%)	3495 (13.23%)	2840 (10.02%)	1448 (7.37%)	1298 (5.46%)	
**Pack-years of cigarettes**	17.83 (26.7)	21.92 (30.04)	18.61 (27.12)	16.25 (25.02)	13.67 (22.56)	<0.001
**Alcohol intake**						<0.001
Never	9920 (10.11%)	2665 (10.09%)	2827 (9.98%)	1963 (9.99%)	2465 (10.38%)	
Former	14233 (14.5%)	4429 (16.76%)	4033 (14.23%)	2578 (13.12%)	3193 (13.44%)	
Current	71245 (72.58%)	18578 (70.31%)	20687 (73.01%)	14556 (74.1%)	17424 (73.34%)	
Unknown	2761 (2.81%)	751 (2.84%)	787 (2.78%)	546 (2.78%)	677 (2.85%)	
**Family history of lung cancer**						<0.001
Yes	10268 (10.46%)	2858 (10.82%)	2960 (10.45%)	1986 (10.11%)	2464 (10.37%)	
No	85541(87.15%)	22819 (86.36%)	24676 (87.09%)	17240 (87.77%)	20806 (87.57%)	
Possible	2350 (2.39%)	746 (2.82%)	698 (2.46%)	417 (2.12%)	489 (2.06%)	
**Family history of any cancer**						0.001
Yes	54835 (55.86%)	14624 (55.35%)	15729 (55.51%)	10952 (55.76%)	13530 (56.95%)	
No	43324 (44.14%)	11799 (44.65%)	12605 (44.49%)	8691 (44.24%)	10229 (43.05%)	
**Diabetes**						<0.001
Yes	6568 (6.69%)	1988 (7.52%)	2065 (7.29%)	1260 (6.41%)	1255 (5.28%)	
No	91591 (93.31%)	24435 (92.48%)	26269 (92.71%)	18383 (93.59%)	22504 (94.72%)	

*Two-sided Ps were based on the Kruskal-Wallis test for continuous variables or Chi-square test for categorical variables.

### Association Between DRRD Score and the Incidence of Lung Cancer

**Figure 2:**
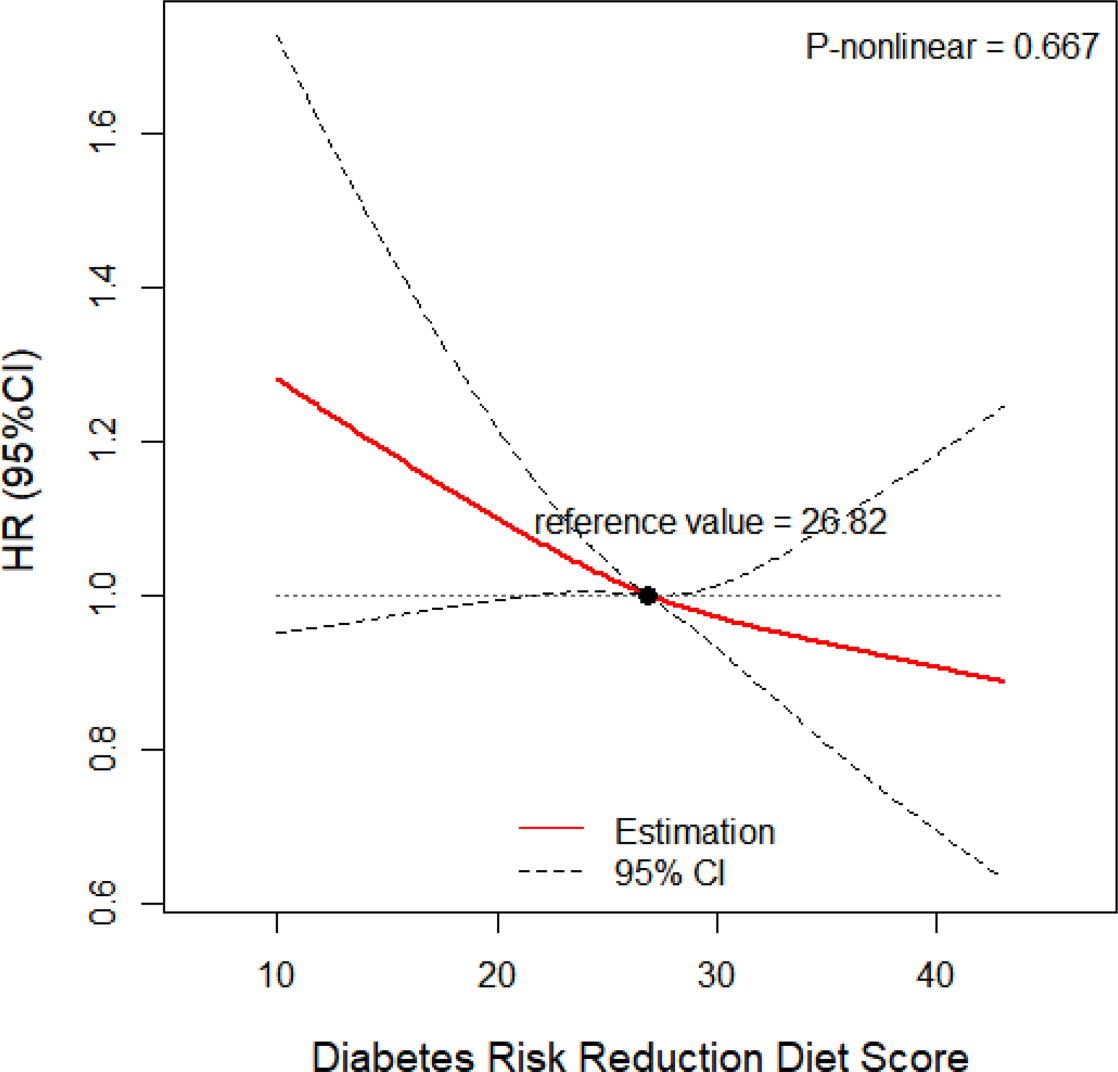
Dose-response relationship between DRRD score and risk of lung cancer adjusted for age (continuous), sex (male or female), BMI (continuous), total energy intake (continuous), family history of lung cancer (yes, no, or possible), marital status (married or not married), race/ethnicity (white or non-white), smoking status (never, former smoker, current smoker), pack-years of cigarettes (continuous), alcohol intake (never, former, current, or unknown), history of diabetes (yes or no) (p for nonlinear= 0.667).

**Figure 3:**
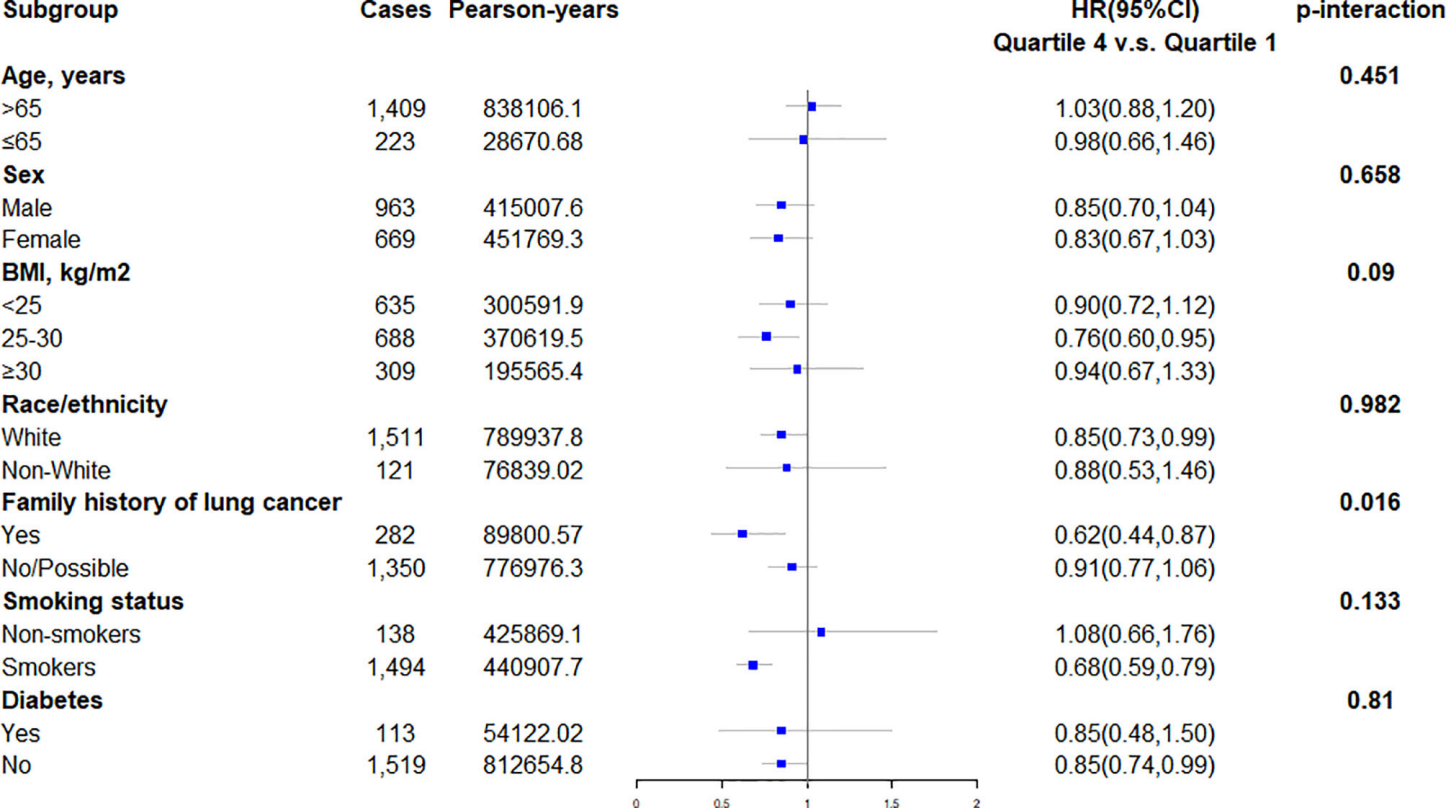
Subgroup analyses evaluating the association between DRRD score and incidence of lung cancer modified by age (>65 vs. ≤65 years old), sex (male vs. female), BMI (>25 vs. 25-30 vs. ≥30 kg/m2), race/ethnicity (white vs. non-white), family history of lung cancer (yes vs. no/possible), smoking status (non-smokers vs. smokers), and history of diabetes (yes vs. no).

The calculated HRs showed there was a trend that higher quartile indicated lower risk of lung cancer unadjusted for covariates (HR_Q4vsQ1_: 0.61; 95% CI:0.53,0.70; p for trend <0.001) and even after adjusted for covariates (HR_Q4vsQ1_: 0.85; 95% CI:0.73,0.98; p for trend =0.036) in all included populations. In the adjusted model, closer examination showed that the inverse trend between higher DRRD score and lung cancer was also present for squamous cell carcinoma (HR_Q4vsQ1_: 0.50; 95% CI:0.34,0.73; p for trend =0.002), but not for adenocarcinoma, large cell carcinoma, or small cell carcinoma (all p for trend>0.05) **(** **Table 2****)**. We employed a restricted cubic spline model to explore the dose-response relationship between DRRD score and lung cancer risk. A linear association between DRRD score and lung cancer was found in the restricted cubic spline model with three knots at the 10th, 50th, and 90th percentiles (reference value=26.82) (p for nonlinear= 0.667) **(** **Figure 2****)**. The risk of lung cancer decreased with the increase of DRRD score. In the subgroup analyses, the association of DRRD score with the risk of lung cancer modified by age, sex, BMI, race/ethnicity, family of lung cancer, smoking status, and history of diabetes was evaluated. The inverse association between DRRD score with the incidence of lung cancer was more pronounced in participants who had a clear family history of lung cancer (p for interaction=0.016), though age, sex, BMI, race/ethnicity, smoking status, or history of diabetes did not significantly affect the observed association between DRRD score and lung cancer risk (all p for interactions>0.05) **(** **Figure 3****).** The sensitivity analysis showed the HRs did not change significantly by excluding participants with extreme energy intake (>4000 kcal/day or <500 kcal/day), with extreme BMI (top 1% and bottom 1%), with diabetes, or with a follow-up less than 2 years, indicating a good robustness of the association between DRRD score and the incidence of lung cancer **(** **Table 3****)**.

**Table 2:** HRs of the association between DRRD score and the incidence of lung cancer and sub-histology types.

DRRDs Quartiles	Number of cases	Unadjusted HR (95%CI)	Adjusted HR (95%CI)*
**Overall**			
Quartile 1 (9-23)	555	1.00 (reference)	1.00 (reference)
Quartile 2 (24-27)	482	0.80 (0.71,0.90)	0.89 (0.79,1.01)
Quartile 3 (28-30)	281	0.67 (0.58,0.77)	0.83 (0.72,0.96)
Quartile 4 (31-45)	314	0.61 (0.53,0.70)	0.85 (0.73,0.98)
p-trend		<0.001	0.036
**Adenocarcinoma**			
Quartile 1 (9-23)	177	1.00 (reference)	1.00 (reference)
Quartile 2 (24-27)	162	0.84 (0.68,1.04)	0.91 (0.73,1.12)
Quartile 3 (28-30)	115	0.86 (0.68,1.08)	0.99 (0.78,1.26)
Quartile 4 (31-45)	140	0.86 (0.69,1.07)	1.07 (0.85,1.35)
p-trend		0.354	0.557
**Squamous cell carcinoma**			
Quartile 1 (9-23)	124	1.00 (reference)	1.00 (reference)
Quartile 2 (24-27)	107	0.80 (0.61,1.03)	0.93 (0.71,1.20)
Quartile 3 (28-30)	50	0.53 (0.38,0.74)	0.71 (0.51,1.00)
Quartile 4 (31-45)	31	0.32 (0.22,0.46)	0.50 (0.34,0.73)
p-trend		<0.001	0.002
**Large cell carcinoma**			
Quartile 1 (9-23)	19	1.00 (reference)	1.00 (reference)
Quartile 2 (24-27)	14	0.68 (0.34,1.36)	0.86 (0.43,1.72)
Quartile 3 (28-30)	5	0.35 (0.13,0.94)	0.51 (0.19,1.39)
Quartile 4 (31-45)	11	0.64 (0.30,1.33)	1.06 (0.49,2.30)
p-trend		0.182	0.549
**Small cell carcinoma**			
Quartile 1 (9-23)	80	1.00 (reference)	1.00 (reference)
Quartile 2 (24-27)	78	0.90 (0.66,1.23)	1.03 (0.75,1.41)
Quartile 3 (28-30)	31	0.51 (0.34,0.77)	0.71 (0.47,1.08)
Quartile 4 (31-45)	38	0.51 (0.35,0.76)	0.87 (0.58,1.30)
p-trend		<0.001	0.319

*Adjusted for age (continuous), sex (male or female), BMI (continuous), total energy intake (continuous), family history of lung cancer (yes, no, or possible), marital status (married or not married), race/ethnicity (white or non-white), smoking status (never, former smoker, current smoker), pack-years of cigarettes (continuous), alcohol intake (never, former, current, or unknown), history of diabetes (yes vs. no).

**Table 3:** Sensitivity analyses to assess the robustness of the association between DRRD score and lung cancer.

	Excluding participants	Number of cases	Person-years	Adjusted HR (95%CI)*
Primary analysis	0	1632	866776.8	0.85 (0.73,0.98)
Excluding participants with extreme energy intake	1414	1594	854674.8	0.86 (0.74,0.99)
Excluding participants with extreme BMI	1930	1600	850434.9	0.85 (0.73,0.98)
Excluding participants with diabetes	6568	1519	812654.8	0.85 (0.74,0.99)
Excluding participants with a follow-up less than 2 years	1608	1336	865107.1	0.84 (0.72,0.99)

*Adjusted for age (continuous), sex (male or female), BMI (continuous), total energy intake (continuous), family history of lung cancer (yes, no, or possible), marital status (married or not married), race/ethnicity (white or non-white), smoking status (never, former smoker, current smoker), pack-years of cigarettes (continuous), alcohol intake (never, former, current, or unknown), history of diabetes (yes vs. no). Note: extreme energy intake was defined as >4000 kcal/day or <500 kcal/day; extreme BMI was defined as top 1% or bottom 1% in the included population.

## Discussion

This study explores the association between DRRD score and lung cancer in a large population. The results show an inverse association between DRRD and the risk of lung cancer, even after adjusting for confounding factors. Though DRRD score has been developed to prevent T2D, the significant inverse association was detected among the American population. The dose-response analyses also present a declining linear tendency of the risk of lung cancer with DRRD score, indicating the risk of lung cancer may change in a parallel manner with the change of DRRD score. The sensitivity analysis suggests the significant relationship between DRRD score and the incidence of lung cancer is robust by excluding outliers.

Previous studies indicated participants with diabetes are more prone to develop cancers (12, 20) and insulin resistance has been suggested to be associated with increased lung cancer risk (10, 11). Hyperinsulinemia and hyperglycemia may accelerate the biological aging progress (12, 15) and stimulate cellular signaling pathways associated with growth factor-dependent cell proliferation and cancer development (21, 22). Moreover, insulin increases the activity of insulin-like growth factor-1 involved in tumor initiation and progression (23, 24). These processes are likely affected by the DRRD score, which has been suggested to be inversely associated with not only T2D risk but also cancers (8, 25, 26). In addition, higher intake of fiber (27, 28), nuts (29), coffee (30, 31), polyunsaturated fat (32), and fruits (33) are associated with a lower level of inflammation (29, 34, 35), but high GI diet (36), trans fatty acids (37), SSBs (38), and red and processed meats (39) are positively associated with inflammation (40, 41). Namely, all the component parts of DRRD are closely related to chronic inflammation, which is also involved in tumorigenesis (12, 15). We hypothesize the inverse association between DRRD score and the incidence of lung cancer is possibly because of the ease of chronic inflammation, hyperinsulinemia, and insulin resistance. Here, people are encouraged to adopt a healthier dietary habit and have higher intake of cereal fiber, nuts, coffee, fruits, and polyunsaturated fat, which indicates a higher DRRD score; but have less intake of a high GI diet, trans-fat, SSBs/fruit juices, saturated fat and red, processed meats, which indicates a lower DRRD score.

Interestingly, we found an even stronger inverse association between DRRD score and the incidence of squamous cell carcinoma, but not the incidence of adenocarcinoma, large cell carcinoma, or small cell carcinoma. Previous studies also suggested the inverse associations with dietary fiber, yogurt, fruits, and vegetables consumption, even some dietary habit index including Alternative Healthy Eating Index-2010, the alternate Mediterranean Diet score, the Dietary Approaches to Stop Hypertension score, and the Dietary Inflammatory Index were more evident for squamous cell carcinoma than other histological cell types (4, 42). The reason is not exactly clear. In a previous study, among the main histological types of lung cancer, squamous cell carcinoma is the most strongly associated with smoking, which increases the risk of lung cancer in part through its pro-oxidant properties (43). We hypothesize that the strong association between DRRD and the incidence of squamous cell carcinoma is related to the mitigation of chronic inflammation. More research should be conducted to detect the reasons behind this.

In subgroup analyses, the magnitude of associations did not change appreciably stratified by age, sex, BMI, race/ethnicity, smoking status, or history of diabetes. However, the significant association between DRRD score with the incidence of lung cancer was more pronounced in participants who had a clear family history of lung cancer, with an adjusted HR of 0.62 (95% CI 0.44-0.87). Consistently, dietary factors were previously found to be associated with cancer risk among individuals with a family history of certain cancers (44, 45). Previous evidence suggests gene expression may be changed by dietary factors *via* epigenetic mechanisms (46). For instance, the consumption of a ‘‘Western-type’’ diet was found to functionally alter the hepatic gene expression by affecting histone polyacetylation and reducing short-chain fatty acids (47). Similarly, the result of our stratified analysis in family history of lung cancer could be attributed to the heritability of genes related to lung cancer susceptibility. The susceptible genes of individuals with a family history of lung cancer may be regulated by dietary habits in the long run. Further research is needed to explore whether the association between DRRD score and lung cancer risk could be explained by the regulation of genes related to both diabetes and lung cancer. The subgroup analyses also showed the opposite association between DRRD and lung cancer risk in former/current smokers and nonsmokers. Because smoking increases the level of inflammation in the human body and thus raises the risk of lung cancer (48), the inverse association between DRRD and lung cancer might be more evident in smokers since adherence to DRRD indicated the alleviation of inflammation.

Of note, this study has remarkable strengths. This prospective large-scale study explores the association between DRRD score and the incidence of lung cancer for the first time in a large population. In this study, the follow-up time was calculated from the completion of DHQ. Because the average time from randomization to the completion of DHQ was approximately 3 years, the actual time for observation in this cohort was far more than 8 years. An appropriate observation time ensured the event to be obtained. In addition, the results were adjusted for a wide range of potential confounding factors, though we could not exclude the possibility that more unmeasured residual confounders might influence the observed association. Furthermore, the sensitivity analyses were conducted to assess the robustness of the association between DRRD score and lung cancer. Moreover, we interestingly found the inverse association between DRRD score and the incidence of lung cancer was more pronounced in participants with a clear family history of lung cancer, though the reason is still unclear exactly.

However, shortages exist in this study. First, the dietary habits may change during the long follow-up, but using a baseline diet only to evaluate the dietary intake generally yielded weaker associations with the incidence of disease than using the cumulative dietary intake (49). Second, the information of self-reported DHQ may not be precise enough because of the huge contents. Third, some subgroups included too small numbers of outcome events to allow sufficient statistical power to observe significant associations and detect potential interactions between DRRD score and stratified factors. Fourth, there were around 36.6% participants (56,728 of 154,887) excluded because they had invalid BQ or DHQ, or were diagnosed with cancer before completing the DHQ. Selection bias might exist in this process though the baseline information could not be compared between the excluded group and included one due to several missing values.

## Conclusion

In conclusion, this study shows an inverse association between DRRD score and risk of lung cancer. People are encouraged to have more cereal fiber, nuts, coffee, fruits, diet with higher ratio of polyunsaturated to saturated fat, which indicates higher DRRD score; but have less intake of high GI diet, trans-fat, SSBs/fruit juices, and red and processed meats, which indicates a lower DRRD score. Further studies should be scheduled to confirm the association.

## Data Availability Statement

The original contributions presented in the study are included in the article/supplementary material. Further inquiries can be directed to the corresponding authors.

## Ethics Statement

This study has been approved by the United States NCI (CDAS project “PLCO-800”). The written informed consent to participate in the study was provided by each participant, and the study protocol was approved by the Institutional Review Board of the United States NCI (https://biometry.nci.nih.gov/cdas/plco/). The participants provided their written informed consent to participate in this study.

## Author Contributions

YZ: Conceptualization, methodology, software, formal analysis, data curation, writing - original draft. GZ: Conceptualization, methodology. MZ: Visualization, investigation, validation. LC: Visualization, investigation. HW: Writing - review and editing, supervision. FL: Writing - review and editing, supervision. All authors contributed to the article and approved the submitted version.

## Funding

This work was supported by National Natural Science Foundation of China grant (NSFC No.32070764; No.81800087); 1.3.5 project for disciplines of excellence, West China Hospital, Sichuan University (ZYJC18021); Sichuan Science and Technology Program (No.2021YFQ0030).

## Conflict of Interest

The authors declare that the research was conducted in the absence of any commercial or financial relationships that could be construed as a potential conflict of interest.

## Publisher’s Note

All claims expressed in this article are solely those of the authors and do not necessarily represent those of their affiliated organizations, or those of the publisher, the editors and the reviewers. Any product that may be evaluated in this article, or claim that may be made by its manufacturer, is not guaranteed or endorsed by the publisher.
